# How Polypurine Tract Changes in the HIV-1 RNA Genome Can Cause Resistance against the Integrase Inhibitor Dolutegravir

**DOI:** 10.1128/mBio.00006-18

**Published:** 2018-04-10

**Authors:** Atze T. Das, Ben Berkhout

**Affiliations:** a Laboratory of Experimental Virology, University of Amsterdam, Amsterdam, The Netherlands; Medical School, University of Athens

**Keywords:** HIV-1, drug resistance evolution, drug resistance mechanisms, integrase, reverse transcription

## LETTER

Malet et al. recently reported a novel mechanism for HIV-1 resistance against the integrase inhibitor dolutegravir (1). Whereas integrase inhibitors usually select resistance mutations in the targeted integrase enzyme, they reported the selection of mutations in the 3′ polypurine tract (PPT) in an optimized experimental system for *in vitro* virus evolution. The PPT mutations were recloned into a wild-type HIV-1 backbone and demonstrated to cause a high level of dolutegravir resistance. How the observed PPT mutations cause dolutegravir resistance remained unclear, however.

**FIG 1:**
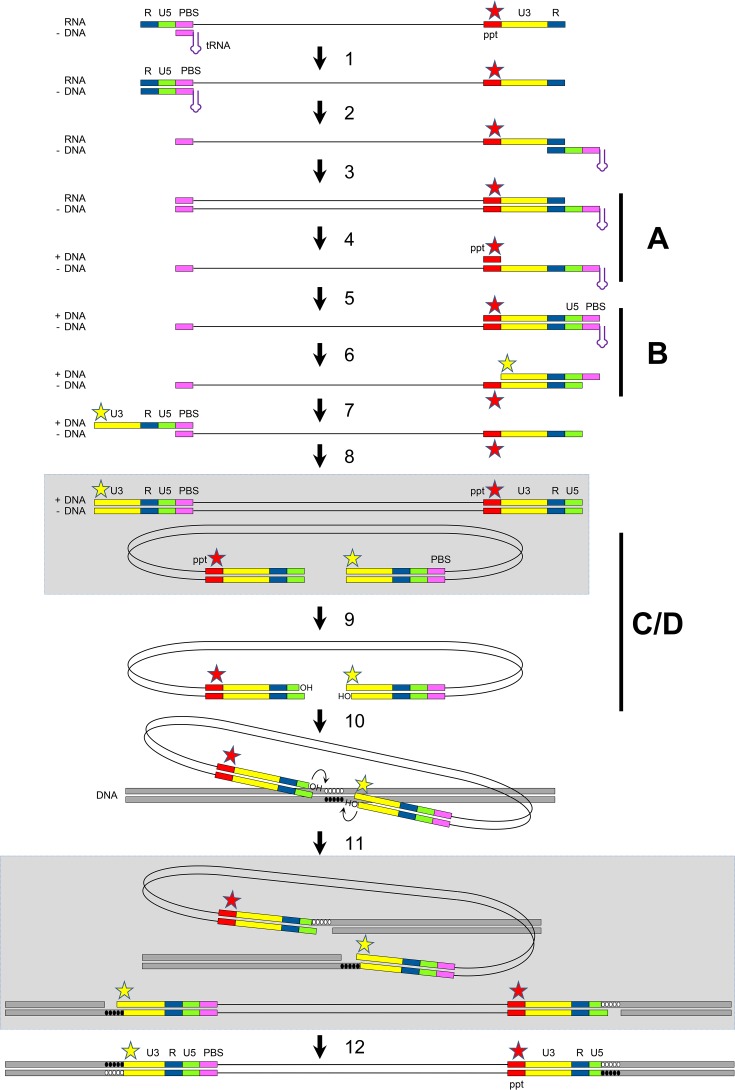
Schematic of the reverse transcription and integration processes. The HIV-1 RNA genome is copied into a dsDNA molecule by the viral RT (steps 1 to 8). This viral DNA is processed and integrated into the cellular genome by the viral integrase (steps 9 to 11) and cellular DNA repair enzymes (step 12). The different steps are described in more detail in the text. The red star marks mutations in the PPT. the yellow star indicates the base pair extension created at the 5′ end of the strong-stop +DNA (step 6) and at the left end of the viral DNA.

The PPT acts as a primer for plus-strand DNA (+DNA) synthesis during the reverse transcription process (Fig. 1, step 5). This process eventually generates the complete HIV-1 double-stranded DNA (dsDNA) copy (step 8) that is integrated into the cellular genome (step 11). To complicate matters, the PPT region overlaps with the viral gene encoding the Nef protein, but a role for this viral protein in the resistance mechanism was dismissed (1). The authors suggested two possible PPT-mediated resistance mechanisms. First, the PPT mutant virus may be able to replicate without DNA integration, but there is no precedent for such a replication strategy among retroviruses. Second, integration of the PPT mutant HIV-1 DNA may proceed in an integrase-independent manner but this also represents a rather unlikely scenario (2).

We here propose an alternative explanation for this unusual resistance mechanism that is based on the coupling of events during the complicated reverse transcription and integration processes (Fig. 1). Briefly, the PPT mutations alter RNase H processing during the reverse transcription process, which leads to the generation of an HIV-1 copy DNA with a modified 5′ end (here, the left end). This modified DNA end may prevent the binding of dolutegravir to the integrase-viral DNA complex, such that integration becomes dolutegravir resistant. To explain how changes in the centrally localized PPT domain affect the left end of the viral DNA, one has to dive deep into details of the reverse transcription process.

The HIV-1 RNA genome is copied into copy DNA by the viral reverse transcriptase (RT) that starts from the cellular tRNA^lys3^ primer annealed to the primer-binding site (PBS; step 1). The primer is extended up to the 5′ end of the RNA, yielding a strong-stop minus-strand DNA (−DNA). Upon degradation of the copied repeat (R)-U5 RNA fragment through RNase H activity within the RT complex, the strong-stop −DNA fragment is released and reanneals to the complementary 3′ R region in the first strand transfer process (step 2). When −DNA synthesis is continued, the PPT sequence and upstream viral sequences are copied (step 3). Unlike the other RNA sequences, the PPT resists subsequent RNase H cleavage (step 4), such that a primer for +DNA synthesis is generated. Extension of this 15-nucleotide (nt) PPT primer results in a strong-stop +DNA fragment in which the U3, R, U5, and tRNA^lys3^ (PBS) sequences are copied (step 5). Upon RNase H cleavage of the PPT and tRNA^lys3^ RNA nucleotides (step 6), the +DNA fragment is released and its PBS sequence reanneals to the complementary PBS sequence of the −DNA in the second strand transfer process (step 7). Continued −DNA and +DNA synthesis leads to the production of a full-length dsDNA (step 8) that is ready for integration into the host cell genome. To ease visualization of the subsequent integration process, this intermediate is also shown in the circular format in Fig. 1. The viral integrase enzyme processes both 3′ ends of this HIV-1 DNA, removing a dinucleotide and liberating 3′ hydroxyl groups attached to 5′-CA-3′ dinucleotides (step 9). Upon binding of the integrase-viral DNA complex to the cellular DNA, the enzyme uses these hydroxyl groups as nucleophiles to cut the cellular DNA in a 5-nt staggered fashion (step 10) and to join both viral DNA ends to the cellular DNA strands (step 11; also shown in linear format). Finally, gap repair by host DNA repair enzymes occurs rapidly (step 12).

We will explain how PPT mutations can influence the viral DNA product of the reverse transcription process, such that the DNA integration process becomes resistant to the dolutegravir inhibitor. Most of these arguments stem from HIV-1 research, but some basic concepts of the reverse transcription and integration mechanism were revealed for other retroviruses. We will focus on four interlinked decisive steps that are marked A to D in Fig. 1. Step A is PPT processing by RNase H. Mutations in the 6-nt G tract at the 3′ end of the PPT (marked by a red star) in the HIV-1 RNA genome shift the RNase H cleavage site (3, 4). It was proposed that repositioning of the RT polymerase would cause RNase H to cleave the substrate one or a few nucleotides upstream of the normal cleavage site at the PPT-U3 junction, resulting in a shortened PPT primer for subsequent +DNA synthesis. This shift was especially pronounced upon mutation of the second or fifth G residue (5), both of which are well conserved among different retroviruses and known to make specific contacts with amino acids in the RNase H domain that are important for cleavage specificity (6, 7). Intriguingly, these two nucleotides were also found to be mutated in the dolutegravir-resistant virus described by Malet et al. (1). Step B is generation of a modified DNA end. The shortened PPT primer may not affect its role in the priming of +DNA synthesis (step 5), but it will change the 5′ end of the HIV strong-stop +DNA that is generated upon removal of the PPT RNA nucleotides in step 6 (marked by a yellow star). More specifically, one or several extra nucleotides will be added to the 5′ end of this +DNA fragment. After the second strand transfer (step 7) and completion of the reverse transcription process (step 8), a viral DNA is produced with one or a few extra base pairs at the left end. Sequences that are critical for retroviral DNA integration are present at the termini of the viral dsDNA, and extension of the ends by a few base pairs can have a major impact on the integration process (8, 9). Consistent with this idea, Malet et al. (1) did observe a profound (approximately 90%) fitness loss in the PPT mutant virus. Step C is altered integrase binding and dolutegravir resistance. Dolutegravir, like other integrase strand transfer inhibitors, was selected for strong binding of preassembled integrase-viral DNA complexes, thereby competing with the target DNA. The additional base pairs at the left end of the viral DNA may alter the integrase-viral DNA complex and thus prevent dolutegravir binding. A more detailed analysis of how this structural change can cause dolutegravir resistance is hampered by the absence of a crystal structure of the full-length integrase-viral DNA-dolutegravir complex, although several molecular modeling studies based on the available structure of integrase subdomains have been performed (10). Step D is cross talk between the 5′ and 3′ ends of HIV-1 DNA during integration. The novel resistance scenario may suffice for dolutegravir-resistant integration of the modified left end of the viral DNA molecule, but one might expect a normal integrase-viral DNA complex to be formed at the unmodified right end, which would still be able to bind dolutegravir and thus make the virus dolutegravir sensitive. However, retroviral DNA integration takes place in the context of the intasome nucleoprotein complex (11), which contains both viral DNA ends and multiple integrase molecules (12–14). The simultaneous interaction of integrase with both viral DNA ends triggers cooperativity during 3′ processing and the strand transfer process. For example, mutation of conserved sequences at the right end of murine leukemia virus DNA impaired integrase-mediated processing not only at the altered right end but also at the unaltered left end (15). Considering the coordinated activity at both DNA ends in multimeric integrase complexes, we propose that the extended left end will modify the integrase-viral DNA complex in such a way that processing of both ends becomes insensitive to dolutegravir.

On the basis of this PPT model of dolutegravir resistance, we predict that the PPT mutant virus will lead to modification of the left end of HIV-1 DNA, which could simply be tested by sequencing of the integrated viral genome. The proposed structural changes in the integrase-viral DNA complex will likely affect the binding of other inhibitors that bind the integrase-viral DNA complex in a similar but not identical way (10). Malet et al. did, indeed, report such cross-resistance of the PPT mutant virus against raltegravir and elvitegravir (1), although this was not tested for in the recloned virus that carries exclusively the PPT mutations.

The new PPT-mediated dolutegravir resistance model is quite unique in that it requires coupling of the reverse transcription and integration processes to fully comprehend the molecular mechanism. Other complex resistance mechanisms have previously been reported in the literature, e.g., resistance to protease inhibitors conferred by altered cleavage sites in the Gag substrate (16) or by a change in the frameshift signal to compensate for the loss of enzyme function by simply making more of the poorly active enzyme (17).
